# Thermally stable hybrid polyarylidene(azomethine-ether)s polymers (PAAP): an ultrasensitive arsenic(III) sensor approach

**DOI:** 10.1080/15685551.2018.1471793

**Published:** 2018-05-22

**Authors:** Mohammed M. Rahman, Mahmoud A. Hussein, Kamal I. Aly, Abdullah M. Asiri

**Affiliations:** a Chemistry Department, Faculty of Science, King Abdulaziz University, Jeddah, Kingdom of Saudi Arabia; b Center of Excellence for Advanced Material Research (CEAMR), King Abdulaziz University, Jeddah, Kingdom of Saudi Arabia; c Polymer Chemistry Lab. 122, Chemistry Department, Faculty of Science, Assiut University, Assiut, Egypt

**Keywords:** Polyarylidene(azomethine-ether)s (PAAP), diarylidenecycloalkanones, thermal behavior, arsenic (III), electrochemical method, real sample analyses

## Abstract

A new category of thermally stable hybrid polyarylidene(azomethine-ether)s and copolyarylidene(azomethine-ether)s (PAAP) based on diarylidenecycloalkanones has been synthesized using solution polycondensation method. For potential cationic sensor development, a thin layer of PAAP onto a flat glassy carbon electrode (GCE, surface area: 0.0316 cm^2^) was prepared with conducting nafion (5%) coating agent to fabricate a sensitive and selective arsenic (III) [As^3+^] ion in short response time in neutral buffer system. The fabricated cationic sensor was measured the analytical performances such as higher sensitivity, linear dynamic range, detection limit, reproducibility, and long-term stability towards As^3+^ ions. The sensitivity and detection limit were calculated as 2.714 μAμM^−1^cm^−2^ and 6.8 ± 0.1 nM (SNR of 3; 3N/S) respectively from the calibration curve. This novel approach can be initiated a well-organized route of an efficient development of heavy selective arsenic sensor for hazardous pollutants in biological, environmental, and health care fields. Real sample analysis for various environmental sample was performed with PAAP-modified-GCE.

## 1. Introduction

Hybrid materials have been attracted researchers from chemistry and engineering back ground last decade for the fascinating properties related to the combination of both inorganic and organic building blocks via blending or covalent bonding [1]. Amazing and interesting new behaviors have been generated due to the combination of two or more building blocks through the mechanical blending and bonding interaction. Hybrid polymers are a well-known class of polymers that can be designed by carrying two or more different functional groups which related to specific groups of normal types of polymers such as polyamines, polyesters, polyketones, polyethers, polyurethanes, polystyrene, PMMA, polysilazane, polyacrylamide, polyarylidenes, etc. Last few decades, many research works related to the synthesis and characterization of different types of hybrid polymers have been reported [2–15]. Two types of hybrid polymers are reported till now, the first type is organic to organic hybridizations while the second type is inorganic to organic hybridizations. Moreover, hybrid polymers are considered as one of the most popular types of synthetic polymers and copolymers in recent years due to of their new characteristics and specifications that are completely different from both original polymers characterizations. Besides that, the properties of hybrid polymers are also investigated by the chemical capability of the singular components and their way of interaction [16,17]. The target hybrid aromatic polyarylidene(azomethine-ether) is an important type of such polymers. These polymers and copolymers can carry characters of features of components such as polyarylidenes, polyazomrthines, and polyethers.

Polyazomethines base on aromatic or heteroaromatic nucleus have been comprehensively examined in the literature long time ago due to its fantastic advantages. These include:, nonlinear optical properties, semiconducting properties, high thermal & mechanical stabilities over wide range of applications, environmentally stable and a moderate antibacterial effect. In addition to polyazomethines have a great ability to change its structure onto complex formation with different metal ions and protonated form with different acids [18–20]. Polyazomethines can easily fabricated as convenient materials for electronic devices and micro chips. Despite all of these above-mentioned features, polyazomethines still suffer from high melting temperatures and pour solubility in common organic solvents. Such problems make them obstinate for industrial diversion using traditional methods [21–23]. Polyarylidenes are another class of synthetic polymers that can be produced through the formation of arylidene linkage in the polymers main chains. These kind of polymers almost have enjoyable properties which can be applied in a variety of industrial fields. Adequate number of arylidene polymers are found in the literature holding conducting ability, liquid crystal properties, inhibitive corrosion protection, thermal stabilities, attractive morphology, biological screening in some cases and other properties [24–32].

So far, aromatic polyethers are very common high performance class of synthetic polymers which can be easily prepared through the formation of aryl ether linkage as a corner stone in the polymer chain as well as the new ether bond can also treated as the main polymer formulation response. As reported aromatic nucleophilic substitution reaction is the common procedure that extremely applied for such polymerization. Aromatic dihalide molecules invigorated by a high electron deficiency groups are chaired with dihydroxy molecules in order to form the targeted polyethers [33,34]. The presence of ether bonds into the macromolecular back bone with aromatic substituents afford polymers easily treated. Consequently, the polymers display superior melt processing behaviors, solubility, adhesion, hydrolytic stability and thermal oxidative. Furthermore, it can also show moderate high glass transition temperatures and tough mechanical properties as well as liquid crystalline properties which may lead them to be applied in microelectronics fabrications and other applications [35–37]. Polyethers show also great ability for film production that can be used in ulta-filtration membranes gas separation and aerospace vehicles [38].

Arsenic pollution affects regions in all corners of the world. Among others, contaminated areas include Argentina, Bangladesh, China, India, Mexico, Myanmar, Nepal, Pakistan, Vietnam, and parts of the USA [39]. Specifically, contamination of aquifers in Bangladesh is deemed most serious; consequently, a great deal of research efforts and field studies concerning arsenic contamination and mitigation have been focused on this region. Arsenic poisoning incurred from chronic exposure to high levels of arsenic is referred to as arsenicosis. Symptoms of this condition include skin lesions and hard patches on the palms of hands and soles of feet; skin and internal organ cancers; diseases of blood vessels in the legs; and also diabetes, high blood pressure, and reproductive disorders [40]. The current World Health Organization (WHO) maximum contamination limit (MCL) for arsenic in drinking water, defined in 1993, is 10 μg/L [41]. In solution, arsenic is readily converted from one species to another via chemical and biological redox pathways. Since arsenic speciation determines both its bioavailability and its potency as a toxin, there is much interest in the literature for speciation studies of arsenic. Generally speaking, these studies are difficult and expensive to carry out, and so they are not ‘first response measures’[42]. Various nanostructure materials modified electrode has been used to enhance the sensing performance of detection to As(III) which has an obvious connection with skin pigmentation and thickening and various types of cancers of skin, lungs, bladder, kidney, etc [43]. Sensors based on organic molecules, DNA, oligonucleotide, and protein have been developed and tested for heavy metal ions. But many of these techniques have limitations such as elevated working temperatures, lower-water solubility, nitrogen-purge buffer, time consuming, and uneconomical that cause inconvenience for As detection in biological, and environmental samples. So it is necessary to develop a simple, sensitive, and reliable technique for the identification of toxic metallic ions for environmental safety, food quality control, protection, and human being health [44]. At present, development of detection methods such as capillary electrophoresis, fluorimetry, gas and liquid chromatography, mass spectrometry, and spectrophotometry are usually consistent and perceptive, but possess limitations, expensive including time consuming and extraction, and requiring pre-concentration steps which increase the risk generation of other hazardous byproducts, and sample loss [45]. Electrochemical sensing of toxic As molecules represents a promising approach that can be used to complement already existing techniques owing to collective features such as low cost, easy instrumentation, high selective and sensitive, and significant for miniaturization [46]. Arsenic is a toxic substance, which caused various cancers and other serious diseases after long-term exposure [47]. Generally, As contamination in drinking water and groundwater is becoming a threat to global health, and as many as 140 million people worldwide may have been exposed to drinking water with As contamination levels higher than the World Health Organization’s guideline of 10 ppb [48]. As(III) is one of the most harmful substances in water to human health, and its toxicity is at least as 60 times as of As(V) or organic arsenic compounds [49]. Very recently, different analytical techniques for the detection of As(III) at the trace level have been developed, such as high performance liquid chromatography (HPLC), atomic fluorescence spectrometry (AFS), inductively coupled plasma-mass spectrometry (ICP-MS)  [47, 50]  etc. Although these analytic methods might be achieved low detection limits, but still they have some limitations such as expenses, time consuming and high skill requirements for operation. Thus, there is still a necessity to develop a simple, fast and highly sensitive analytic technique for the detection of As(III) in environment. Toxic As^3+^ ion is generally severe to health and environment, therefore it is immediately required the detection by using a reliable cationic-sensor method with PAAP/GCE. The As^3+^ ion by thin PAAP films on GCE was prepared and studied in details of the cationic sensors by electrochemical approaches. The easy-coating method for the construction of PAAP thin-film within binding-agents is executed for preparation of films onto GCE. In this approach, PAAP fabricated films with conducting binders (5% nafion) was utilized towards the target As^3+^ analytes using reliable Current-vs-Voltage method. It was confirmed that the fabricated cationic sensor is unique and noble research work for ultra-sensitive recognition of As^3+^ ions with PAAP/GCE in short response-time.

## 2. Experimental

### 2.1. Reagents and solvents

4,4`-oxo-bis(4``-aminophenylene) from (95%, BDH) was used without purification. 4-bromobenzaldehyde and 4-chlorobenzaldehyde from (95% & 97%, Fluka) and also were used without purification. Cyclohexanone, cyclopentanone, p-hydroxy benzaldehyde and vanillene from (99%, 99%, 98% and 95%, Merck). Anhydrous potassium carbonate from (Aldrich). DMSO analytical grade (99%, Sigma Aldrich). All other reagents used were of high purity and were further purified as reported in literature [50]. Analytical grade of Al_2_(SO_4_)_3_, AuCl_3_, AsCl_3_, Ba(NO_3_)_2_, CaCl_2_, CdSO_4_, Ce(NO_3_)_2_, Co(NO_3_)_2_, MgCl_2_, SbCl_3_, SnCl_2_, YNO_3_, ZnSO_4,_ NaH_2_PO_4_, Na_2_HPO_4_, and nafion (5% ethanolic solution) were purchased from Sigma Aldrich, and used without further purification. Stock solution of As^3+^ ions solution (1.0 M) was prepared from the purchased chemicals. I-V method was conducted to detect As^3+^ ion at a selective point using the fabricated PAAP/GCE by Keithley electrometer (6517A, USA). [Caution! Arsenic is toxic. Only a small amount of this material had been used to prepare the required solution and handled with care.]

### 2.2. Instrumentation

All melting points reported for the monomers, pre-monomers and model compound are uncorrected and determined on a Gallen-kamp Melting Point apparatus with a digital thermometer type MFB-595-010M. Elemental analyses were estimated by an Elemental Analyses system GmbH, VARIOEL, V_2.3_ July 1998 CHNS Mode. IR spectra were determined on IR-470, Infrared spectrophotometer, Shimadzu using the KBr pellet technique. Room temperature ^1^H-NMR spectra were carried out on a varian EM-390-NMR (90 MHz) spectrometer and a GNM-LA 400-MHz NMR spectrophotometer using DMSO or CDCl_3_ as deuterated solvents and in the presence of TMS as an internal reference. Mass spectra were investigated on a Jeol JMS600 mass spectrometer. I-V method (two electrodes composed onto fabricated GCE) was measured for toxic arsenic ions for PAAP/GCE by using Keithley-Electrometer from USA.

### 2.3. Characterization techniques

(0.5% w/v) of polymer solutions in DMSO at 30 ºC were investigated in order to measure the inherent viscosities using an Ubbelohde suspended level viscometer. Solubility character of polymers and copolymers was determined at room temperature using 0.02 g of polymer in 3–5 ml of solvent using DMSO, DMF, THF, DMA, CHCl_3_: CH_3_COCH_3_ (1:1), CH_2_Cl_2_, H_2_SO_4_ concentrated and HCOOH as solvents. The X-ray diffractographs of the polymers were carried out with a Philips X-ray PW1710 diffractometer, and Ni – filtered CuKα radiations. Thermogravimetric analysis (TGA) and differential thermal gravimetric (DTG) were obtained in air with TA 2000 thermal analyzer at heating rate of 10°C/min. in air. The morphological features for polymers and copolymers were estimated by a Scanning electron microscope (SEM) using a Jeol- JSM-5400 LV-SEM. The SEM sample was prepared by putting a smooth part of polymer powder on a copper holder and then coating it with a gold-palladium alloy. SEM micrographs were picked up using a Pentax Z-50P Camera with Ilford film. The images obtained using a low dose technique at accelerating voltage of 15kV. Detailed experimental procedures for monomers (***1_a,b_, 2_a,b_ & 3_a,b_*** their sodium salts ***4_a,b_ & 5_a,b_*)**, model compound (***6***) and polymers (***7_a-d_ & 8_a-f_***) synthesis are studied and described elaborately in the Electronic Supplementary Information file (Ω).

### 2.4. Fabrication of GCE with PAAP

Phosphate buffer solution (PBS, 0.1 M) at pH 7.0 is prepared by mixing of equi-molar concentration of 0.2 M Na_2_HPO_4_ and 0.2 M NaH_2_PO_4_ solution in 100.0 mL de-ionize water at room conditions. GCE is fabricated with PAAP using 5% ethanolic nafion solution as a conducting binder. Then it is kept in the oven at 30.0°C for 1 hour until the film is completely dried, stable, and smooth. A cell is assembled with PAAP/Nafion/GCE and Pd-wire as a working and counter electrodes respectively. As received As^3+^ solution (1.0 M) is diluted to make various concentrations (0.5 M ~ 10.0 nM) in DI water and used as a target analyte. The ratio of current versus concentration (slope of calibration curve) is used to calculate the As^3+^ sensitivity. Detection limit is evaluated from the ratio of 3N/S (ratio of Noise×3 vs. Sensitivity) from the linear dynamic range of calibration curve. Electrometer is used as a constant voltage sources for I-V measurement in simple two electrode system. Amount of 0.1M PBS was kept constant in the beaker as 5.0 mL throughout the chemical investigation. The PAAP is fabricated and employed for the detection of As^3+^ ions in liquid phase. I-V response was measured with PAAP/Nafion/GCE film.

## 3. Results and discussion

The main target in this research work is to synthesize and characterize a new class polymers in the form of polyarylidene(azomethine-ether)s and copolyarylidene-(azomethine-ether)s through solution polycondensation method and to shed light on certain characteristics, such as solubility, thermal stability, crystallinity and morphology. This type of polymers can be applied in different high performance applications. However, that needs special characters such as high thermal stability and high *T_g_* as well according to the application applied for selective and sensitive arsenic sensor development by electrochemical approaches. However, these new polymers and copolymers necessitate the synthesis of required monomers as harbingers.

### 3.1. Monomers synthesis

**Figure 1.**
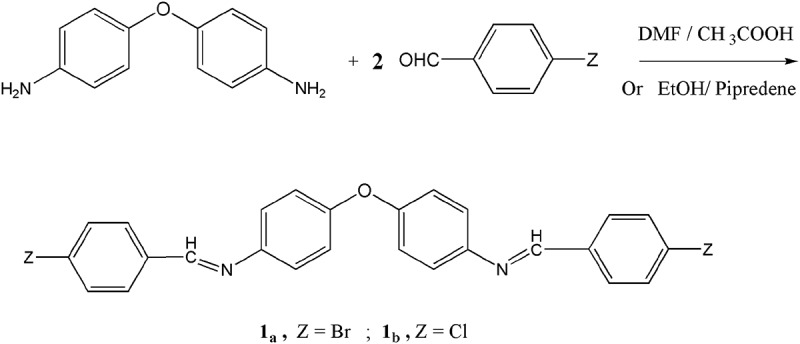
Synthesis of diphenyl ether based monomers **1_a,b._**

Two azomethine monomers **1_a,b_** are synthesized by two different methods, the first method which is considered as acid – catalyzed condensation. In this method 4,4`- diaminodiphenyl ether can easily interact with 4-halobenzaldehyde in 1:2 molar ratio using DMF as a solvent and in the presence of few drops of glacial acetic acid. The second method is accomplished using basic – catalyzed condensation of 4,4`- diaminodiphenyl ether with 4-halobenzaldehyde in absolute ethanol and in the presence of few drops of dry piperidine [51–55] as shown in Figure 1.

**Figure 2.**
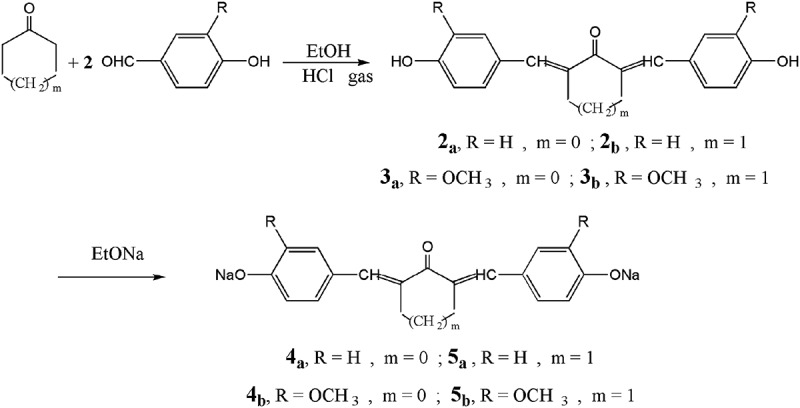
Synthesis of diarylidenecycloalkanone monomers **2_a,b_ & 3_a,b_** and their sodium salts **4_a,b_ & 5_a,b._**

The chemical structure of these azomethine monomers **1_a,b_** are elucidated by both correct elemental and spectral data. The IR spectra showed the disappearance of absorption bands due to primary amino groups, displayed characteristic absorption bands at 1610–620 cm^−1^ due to C = N and absorption band at 1240–1275 cm^−1^ due to C-O-C bonds (ether linkage) ***(cf. figure S1****as an example)*. As well as ^1^H-NMR spectra showed distinctive absorption beaks that attributed to certain groups are found as illustrated in the experimental part (***cf. figure S2****as an example*). Whereas, diarylidenecycloalkanone monomers **2_a,b_** and **3_a,b_** are prepared by forthright condensation of one mole of cycloalkanone with double moles of p-hydroxybenzaldehyde or 4-hydroxy-3-methoxybenzaldehyde in the presence of absolute ethanol and catalytic amount of dry HCl gas as a catalyst. The reaction these monomers with sodium ethoxide afforded readily their sodium salt **4_a,b_** and **5_a,b_** as shown in Figure 2. The structure of these monomers are elucidated by correct elemental and spectral analyses as described in our previous work [56, 57] .

### 3.2. Synthesis of model compound

**Figure 3.**
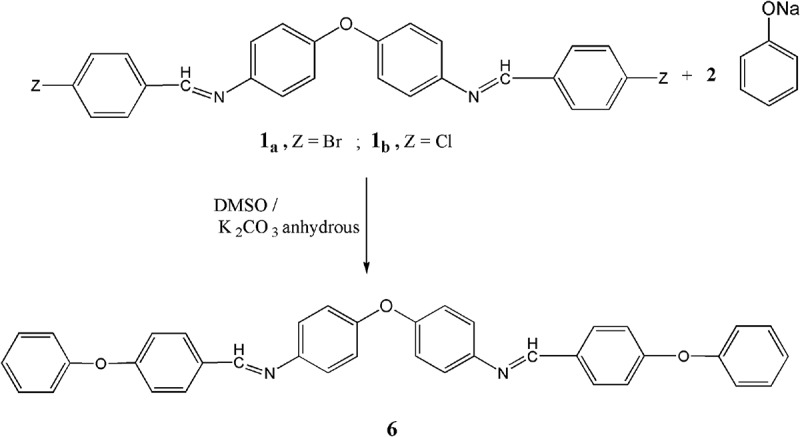
Synthesis of model compound **6.**

Before accomplishing the polymerization process, the aimed model compound **6** are prepared by interacting one mole of 4,4`-oxo-bis(4``-halobenzylideneimino-phenylene) **1_a,b_** with double moles of sodium phenoxide in DMSO as well as in the existence of potassium carbonate anhydrous according to Figure 3. The chemical structure of the resulted model compound **6** is investigated by correct elemental data and spectral analyses. The IR spectrum shows the feature absorption band at 1265 cm^−1^ due to ether connection. Beside, other characteristic peaks are due to other groups. ^1^H-NMR spectrum of model compound **6** show an increase in the aromatic protons and finally the mass spectrum exhibited a molecular ion peak (m/z) in accordance with its molecular structure as shown in the experimental part (***cf. figures S3, S4, S5***).

### 3.3. Synthesis of polyarylidene(azomethine – ether)s 7_a-d_

**Figure 4.**
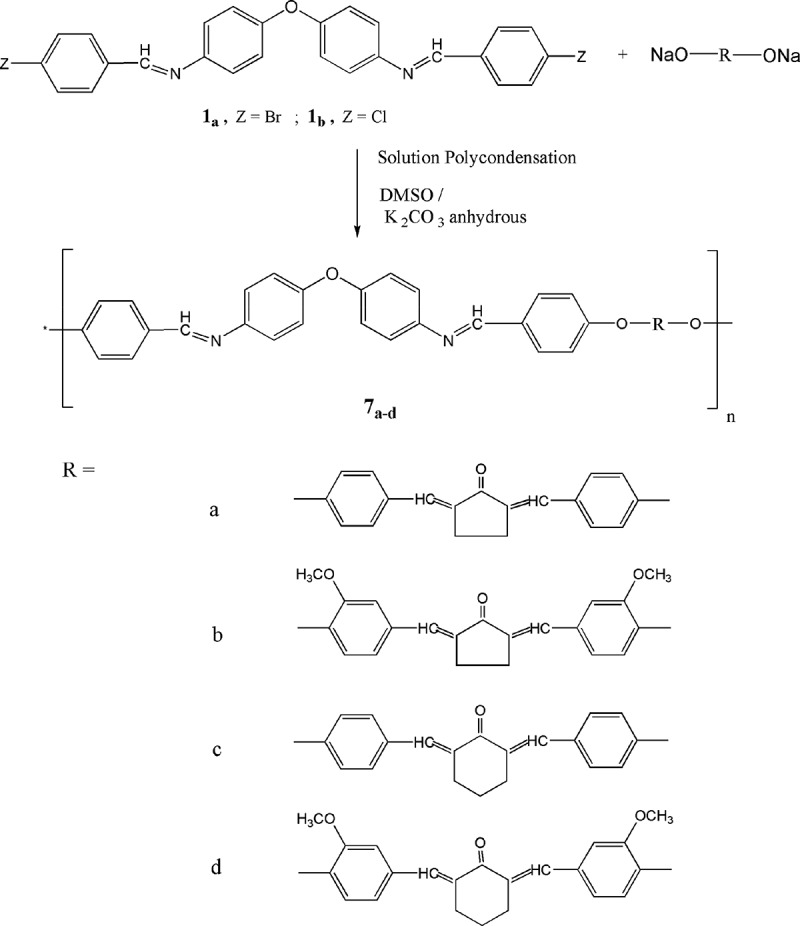
Synthesis of poly(arylidene-ether)s **7_a-d._**

Our aim is to produce a new category of polyarylidene(azomethine – ether)s using solution polycondensation method [25–30]. These new polymers are produced by means of condensation reaction of azomethine monomers **1_a,b_** with diarylidenecycloalkanone monomers **4_a,b_** and **5_a,b_** in DMSO as well as in the presence of potassium carbonate anhydrous [58] as illustrated in Figure 4 . The chemical structure of these new polymers is confirmed by elemental and spectral data. The elemental analyses of all the polymers synchronize with the distinctive periodical units of each polymer; (see experimental part). The elemental analyses of such polymers deviated up to 0.90% from the theoretical amounts.

The IR spectra in KBr pellets for all the formed polymers show the new absorption beaks at 1660–1645 cm^−1^ which due to carbonyl group of the cycloalkanone moiety, absorption beaks at 1250–1275 cm^−1^due to ether linkage. More particularly, other distinctive beaks which are attributed to the other common functional groups present in the polymers structures are also examined such as: at 3090–3030 cm^−1^ for CH stretching of aromatic ring, at 2940–2900 cm^−1^ for CH stretching of aliphatic groups, at 1620–1600 cm^−1^ for C = N azomethine groups (***cf. figure S6****as selected example*).

### 3.4. Synthesis of copolyarylidene(azomethine – ether)s 8_a-f_

**Figure 5.**
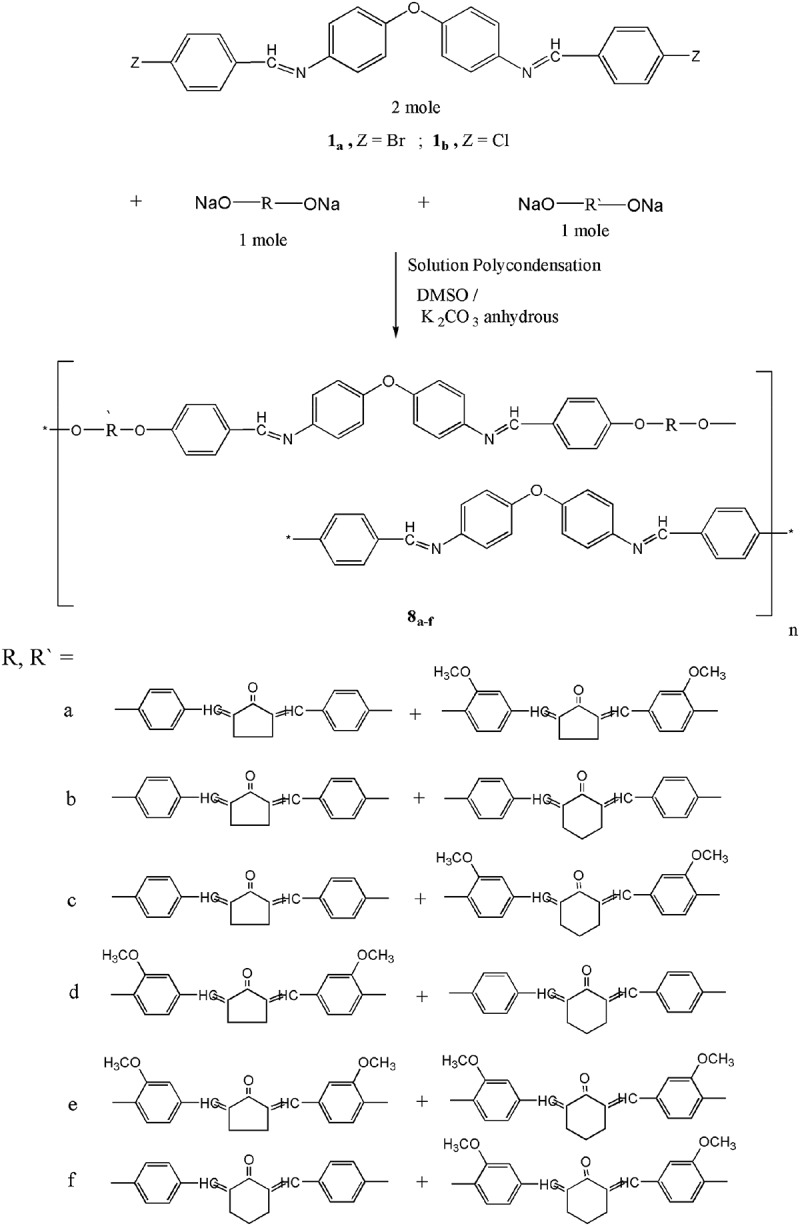
Synthesis of copoly(arylidene-ether)s **8_a-f._**

The titled copolymers are also synthesized by solution polycondensation technique [25–30] using the procedure described in the literature [57] by the interaction of two moles of two varied diarylidenecycloalkanone monomers **4_a,b_** and **5_a,b_** (1:1 molar ratio) together with two moles of azomethine monomers **1_a,b_** in suitable amount of DMSO and in the presence of potassium carbonate anhydrous as well. (cf. Figure 5).

The structure of these polymers is estimated by elemental analyses and spectral data. As well as the elemental analyses of all the copolymers synchronize with the distinctive periodical units of each copolymer; the data are given in the experimental part. Deviation up to 1.1% from the theoretical values is found. The IR spectra in KBr pellets for all the resulting copolymers show the absorption beaks at 1665–1640 cm^−1^ due to carbonyl groups of the cycloalkanone moieties, absorption beaks at 1245–1265 cm^−1^due to ether linkage. at 3040–3020 cm^−1^ for CH stretching of aromatic ring, at 2940–2910 cm^−1^ for CH stretching of aliphatic groups and at 1620–1595 cm^−1^ for azomethine groups (***cf. figure S7****as selected example*).

### 3.5. Polymers and copolymers characterization

Various characterization techniques are applied for the obtained polymers and their corresponding copolymers are determined and the data are discussed below.

Ordinary temperature solubility test for polyarylidene(azomethine–ether)s **7_a-d_** and copolyarylidene(azomethine–ether)s **8_a-f_**, is examined using variety of solvents: DMF, DMSO, DMA, THF, CHCl_3 –_ CH_3_COCH_3_ (1:1), CH_2_Cl_2_, HCOOH and conc. H_2_SO_4_. A standard of 5% (w/v) solution is taken for each sample. It can be clarified from Table 1 that all the polymers and copolymers, are insoluble in alcohols, benzene, and acetone as examples for common organic solvents, but completely soluble in HCOOH and conc. H_2_SO_4_ acids giving an orange to red colors for polymers **7_a-d_** and giving a red to deep red colors for copolymers **8_a-f_**. As it can be also clarified from Table 1 that, the plurality of the polymers and copolymers are also soluble in DMSO exclude polymers, **7_a,b_** which are slightly soluble. Whereas, in polar aprotic solvents such as DMF, DMA it is mentioned that all polymers are partially soluble in both solvents. Whereas, the majority of the copolymer is also soluble in similar solvent except copolymer **8_a_**, which is partially soluble. Moreover, all the polymers as well as copolymers are completely insoluble in CHCl_3 –_ CH_3_COCH_3_ mixture and CH_2_Cl_2_. Furthermore, diarylidenecyclohexanone based polymers are slightly more soluble than on diarylidenecyclopentanone based polymers. Such behavior is clearly noted in aprotic solvents (eg. DMSO, DMF, and THF) than the former polymers. The main reason for this observation is due to the greater flexibility of the cyclohexanone moieties along the chains as detected in our previous studies [13,28]. As well as the solubility character of the synthesized copolymers is slightly higher than the corresponding polymers, which appeared clearly in (DMF, DMA and THF) except copolymer **8_a_** which has partial solubility effect in the same solvents.

**Table 1.** Inherent viscosities and solubility characteristics of poly(arylidene-ether)s 7_a-d_ and their copolymers 8_a-f._

Polymer Number	η_inh_* (dL/g)	DMF	DMSO	DMA	THF	CHCl_3_: CH_3_COCH_3_ (1: 1)	CH_2_Cl_2_	HCOOH	H_2_SO_4_ (Conc)
**7_a_**	**–**	**3**	**3**	**3**	**1**	**1**	**1**	**5**	**5**
**7_b_**	**–**	**3**	**3**	**3**	**1**	**1**	**1**	**5**	**5**
**7_c_**	**0.43**	**3**	**5**	**3**	**1**	**1**	**1**	**5**	**5**
**7_d_**	**0.38**	**3**	**5**	**3**	**2**	**1**	**1**	**5**	**5**
**8_a_**	**0.65**	**3**	**5**	**3**	**2**	**1**	**1**	**5**	**5**
**8_b_**	**0.49**	**4**	**5**	**4**	**2**	**1**	**1**	**5**	**5**
**8_c_**	**0.70**	**4**	**5**	**4**	**2**	**1**	**1**	**5**	**5**
**8_d_**	**0.50**	**4**	**5**	**4**	**2**	**1**	**1**	**5**	**5**
**8_e_**	**0.60**	**4**	**5**	**4**	**2**	**1**	**1**	**5**	**5**
**8_f_**	0.54	4	5	4	2	1	1	5	5

**Table key**. Highly Soluble Soluble Partially soluble Insoluble 5 4 3 2 1 The solubility test has measured at room temperature * Inherent viscosity (**η*_inh_***) was measured at 30°C in DMSO solvent.

The inherent viscosities (η_inh_) of polymers **7_a-d_**, and copolymers **8_a-f_**, are tested in DMSO at 30°C with an Ubbelohde suspended level viscometer. The test is measured only for the completely soluble polymers and copolymers. The inherent viscosity values are calculated according to the following equation:

$$\eta_{inh}=\;[2.3\;log\eta /\eta_{o}]/C$$

The solution concentration C is 0.5g/100 ml, η/η _o =_ relative viscosity (or viscosity ratio). The data are also listed in Table 1. On comparison the inherent viscosity (η_inh_) values for all polymers and copolymers, it was found that the polymer **7_c_** has high viscosity value (0.43 dL/g), and this may be attributed to high molecular weight of this polymer within the series. Whereas, copolymers **7_a,c_** have high viscosity values (0.65, 0.70 dL/g respectively) and this also may be attributed to high molecular weight of these copolymers within the series. Moreover, copolymers **7_b,d_** have low viscosities (0.49 and 0.50 dL/g respectively), which may be attributed to low molecular weight of these copolymers within the series . On comparison between the viscosity values of the resulting polymers and their corresponding copolymers, it was found that, mainly the copolymers have higher viscosity values than the polymers, which may be attributed to the high molecular weight of those copolymers than the corresponding polymers.

**Figure 6.**
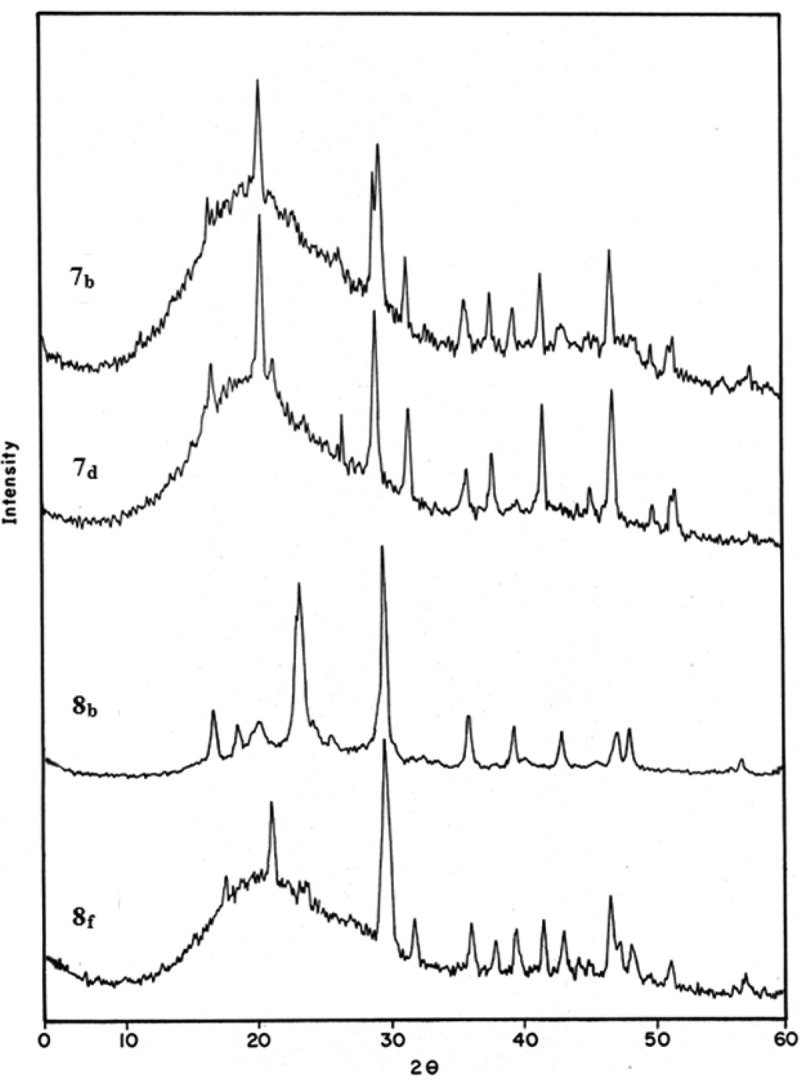
X-ray diffraction patterns of polyarylidene(azomethine – ether)s **7_b,d_** & copolyarylidene(azomethine–ether)s **8_b,f._**

The XRD of selected examples of polyarylidene(azomethine–ether)s **7_b,d_** and copolyarylidene(azomethine–ether)s **8_b,f_** are represented in Figure 6. XRD patterns of polymers **7_b,d_** and copolymer **8_f_** indicate that these polymers are semi-crystalline, where the diffractograms show an amorphous halo beak in the region 2θ = 5–60° and this indicates a low degree of crystallinity. As well as a few reflection beaks that are intermediates between amorphous and crystalline interferences are found. The low crystallinity behavior is exhibited, which might be due to the existence of methoxy groups as pendent groups along the polymer backbone which decrease the crystallinity. Methoxy group adjudicate the chains from each others, cases an efficient obstruction between the repeating units and compel it to unsymmetrical direction [57]. Whereas, XRD patterns of copolymer **8_b_** show that many reflection peaks that are ranging in crystalline phase within the same region of 2θ. It is so easy to say that this copolymer is completely crystalline. This signalizes that there is a major class of structures that are moderate in the ordered states between crystalline and amorphous phases with respect long-range order in the configuration of their atoms and molecules.

**Figure 7.**
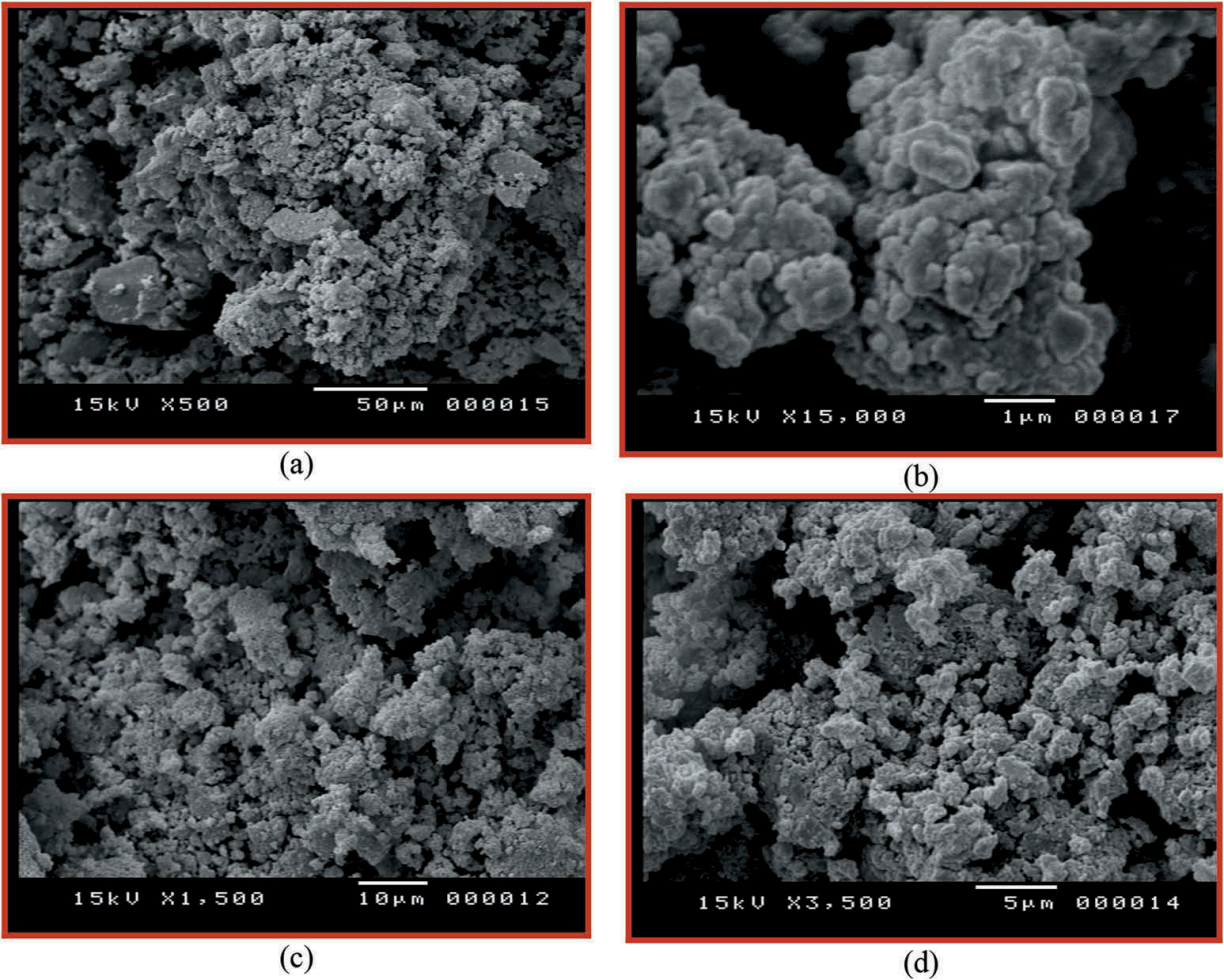
SEM images of polymer **7_c_** surface at magnifications of X (a: X = 500 and b: X = 15,000) and copolymer **8_e_** surface at magnifications of X (c: X = 1500 and d: X = 3500).

The morphology of the synthesized polymers **7_a-d_** and copolymers **8_a-f_**, is tested by scanning electron microscopy (SEM) and the micrographs are given in Figure 7. The samples are prepared as described in the experimental part. The study of selected polymers and copolymers shows that the surface of polyarylidene(azomethine – ether)s **7_c_** (Figure 7a, magnification X = 500) consists of accumulative grain, with higher magnification X = 15,000 (Figure 7b) shows merged spherical particles. Whereas, SEM images show the surface of copolyarylidene(azomethine – ether)s **8_e_** (Figure 7c, magnification X = 1500) consists of porous particles seems to be like spongy shape, with higher magnification X = 3500 (Figure 7d) shows, more porous merged particles with cavity structure.

The thermal properties of the polyarylidene(azomethine–ether)s **7_a-d_**, and copolyarylidene(azomethine–ether)s **8_a-f_**, are determined by TGA, DTG DTA techniques in air at a heating rate of 10°C min^−1^. As mentioned earlier in the results and discussion part and according to the thermal behavior of the new polymers and copolymers. Therefore, it is so important to investigate the thermal degradation for these new polymers and copolymers as well in correlation with their structure in order to explain the better characteristics for such selected applications. TG thermographs of the synthesized polymers and copolymers are illustrated in ***(figures S8, S9 and S10)*** and Table 2 shows the temperatures for 10, 25 and 50% of weight losses. In most cases T_10_ (temperatures for 10% weight loss) are found to be the polymers degradation temperatures (PDT); which means that no significant decomposition are examined before such temperatures [24]. For the polyarylidene(azomethine–ether)s **7_a,c_** the curves show a little weight loss within the range 2–5% (onset at 90°C to 140°C), which is due to loss of imbibed humidity and entrapped solvents. The curves also indicate that the polymers decompose in two steps for polymer **7_c_** and in three steps for polymer **7_a_**. The predictable nature of degradation in the first step is ranged between 245 and 376°C; seems to be slow and count on the origin of these polymers. The degradation in this step is due to a pyrolytic oxidation of carbon-carbon double bonds that come from arylidene moieties followed by cleavage of many other weak bonds. Meanwhile, the second step is ranged between 470 and 575°C and consider as the faster degradation step. The prospective nature of degradation in this step is attribute to cleavage of ether linkage and scission of many other bonds as well. At the end, many burnt segment will be produced as an indicator for the formation of end product. The rate of degradation becomes maximal in the range 530 − 550°C and is almost finished at around 650°C, Polymer **7_a_** show lower T_50%_ value compared with other T_50%_ values of other tested samples which is referred to the lower stability of that polymer. For the copolyarylidene(azomethine–ether)s **8_a,b,d,e_** the thermographs of these copolymers also display a little weight loss within the range 2–4% (onset at 110°C to 180°C), which may be also referred to loss of imbibed humidity and entrapped solvents. The curves also mention that the majority of the copolymers decompose in two stages except copolymer **8_b_** in three steps. The prospective nature of degradation in the first and second steps is ranged between 200 and 370°C and 380 and 570°C respectively. Whereas, the third step for polymer **8_b_** is ranged between 580 and 670°C. No significant changes are found in the decomposition behaviors for these copolymers than previously mentioned polymers; nearly the same explanation is utilized. All the selected polymers and copolymers have PDT in the range of 145 ~ 340°C. Consequently, the results in Table 2 announce that the order of thermal stability of these samples is **7_c_ > 8_d_ > 8_e_ > 8_a_ > 8_b_ > 7_a_**. The abbreviation (FDT) is related to the final decomposition temperature and it is defined as the temperature at which the rate of decomposition is almost finished. So far, the given TG micrographs also display that all the tested polymers and copolymers have very high FDT values which are ranged between 572 and > 650°C. This is of course another good indication for the higher thermal stability for the desired polymers and copolymers even for polymer **7_a_**. Polymer **7_a_** has lower thermally stable values at T_10_, T_25_ and T_50_ while compared with other substituents. Whereas, it has higher FDT value nearly like the other derivatives. Which mean that the decomposition can be completed at very high temperature.

**Table 2.** Thermal behavior of poly(arylidene-ether)s **7_a,c_** and copoly(arylidene-ether)s **8_b,d._**

		Temperature for various percentage decompositions (°C)*			
Polymer Number	PDT_max_*	10%	25%	50%	*PDT_f_* *
**7_a_**	485.8	145	335	483	> 650
**7_c_**	568.4	340	387	558	572
**8_a_**	566.1	323	382	540	650
**8_b_**	574.8	318	374	560	650
**8_d_**	534.4	330	386	570	590
**8_e_**	565.5	327	393	547	> 650

* The values were determined by TGA at heating rate of 10°C min^−1^.

The determination of glass transition temperature *T_g_* by using DTA measurements are well known technique. Big number of synthetic polymers with long chain show distinctive succession of changes when they are heated. DTA curves for polymers **7_a,c_**, and of copolymers **8_d,e_** as selected examples are given in ***(figures S11 and S12***). It can be seen that the majority of these polymers show a broad endothermic peaks become clear above 190°C. Since, the TGA hints does not show thermal decomposition at this temperature except for polymer **7_a_**, which shows a distinct weak glass transition temperature *T_g_* at 57.08°C, which in agreement with its thermal degradation at 10%. Furthermore, polymer **7_c_** shows *T_g_* at 215.56°C and no *T_m_* more has been estimated. It should be noted that the disappearance of *T_m_* of these polymers may be attributed to the rapid decomposition. As well as, copolymer **8_d_** and copolymer **8_e_** show *T_g_* at 194.21°C & 225.19°C and *T_m_* at 397.75°C & *T_m_* at 403.7°C respectively. It is easily noticed from the above results that, all of tested polymers and copolymers have high *T_g_* values which is attributed to the high rigidity of those polymers chains.

### 3.6. Application: detection of As^3+^ ions using PAAP/GCE by I-V method

**Scheme 1.**
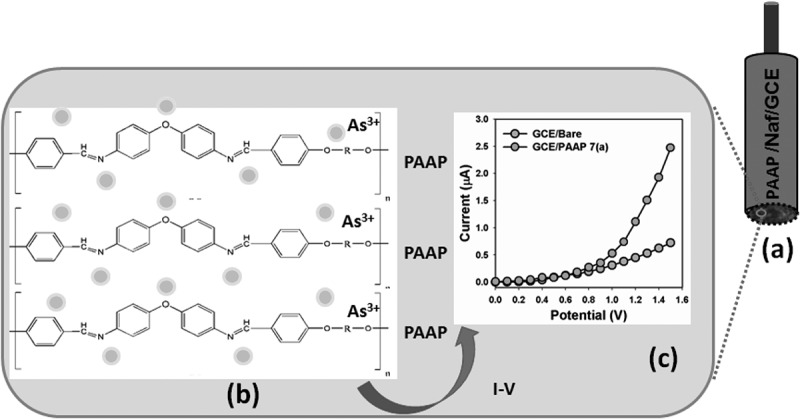
Mechanism of the probable interaction of As^3+^ with PAAP polymer with conducting nafion binders embedded onto GCE. (a) Fabricated GCE electrode, (b) inter- or intra-molecular interactions between lone-pair of nitrogen and oxygen (PAAP) and As^3+^, and (c) current responses in presence of As^3+^ ions by I-V method.

The potential application of PAAP assembled onto GCE as a heavy and toxic arsenic sensor (especially As^3+^ analyte in buffer system) has been investigated for measuring and detecting target As^3+^ ions. The PAAP/GCE sensors have facile advantages such as stability in air, non-toxicity, chemical inertness, electro-chemical activity, simplicity to assemble, ease in fabrication, and chemo-safe characteristics. As in the case of As^3+^ ions sensor, the current response in I-V method of PAAP/GCE considerably changes when aqueous metallic analyte is adsorbed. The PAAP/GCE was applied for fabrication of ionic-sensor, where heavy metallic As^3+^ ion was measured as target analyte. The fabricated PAAP/GCE electrode was put into the oven at low temperature (30.0°C) for 1.0 hrs to make it dry, stable, and uniform the surface totally. I-V signals of As^3+^ ion chemical sensor are anticipated having PAAP/GCE on thin-film as a function of current versus potential. The resultant electrical responses of target As^3+^ ion are investigated by simple and reliable I-V technique using PAAP/Nafion/GCE assembly. The holding time of electrometer was set for 1.0 sec. A significant amplification in the current response with applied potential is noticeably confirmed. I-V characteristic of the PAAP/Nafion/GCE is activated as a function of As^3+^ ions concentration at room conditions, where improved current response is presented (Scheme 1). For a low concentration of As^3+^ ions in liquid medium, there is a smaller surface coverage of As^3+^ ions molecules on PAAP/Nafion/GCE film and hence the surface reaction proceeds steadily. By increasing the As^3+^ ions concentration, the surface reaction is increased significantly (gradually increased the response as well) owing to surface area (assembly of PAAP/Nafion/GCE) contacted with As^3+^ ions molecules (Scheme 1a). Further increase of As^3+^ ions concentration on PAAP/Nafion/GCE surface, it is exhibited a more rapid increased the current responses, due to larger area covered by As^3+^ ions and the interaction of the nitrogen and oxygen containing functional groups (Scheme 1b). The molecular interaction could be approaches as inter-molecular and intra-molecular interactions of the PAAP polymer chains. Usually, the surface coverage of As^3+^ ions on PAAP/binders/GCE surface is reached to saturation, based on the regular enhancement of current responses, which shown in Scheme 1c.

**Figure 8.**
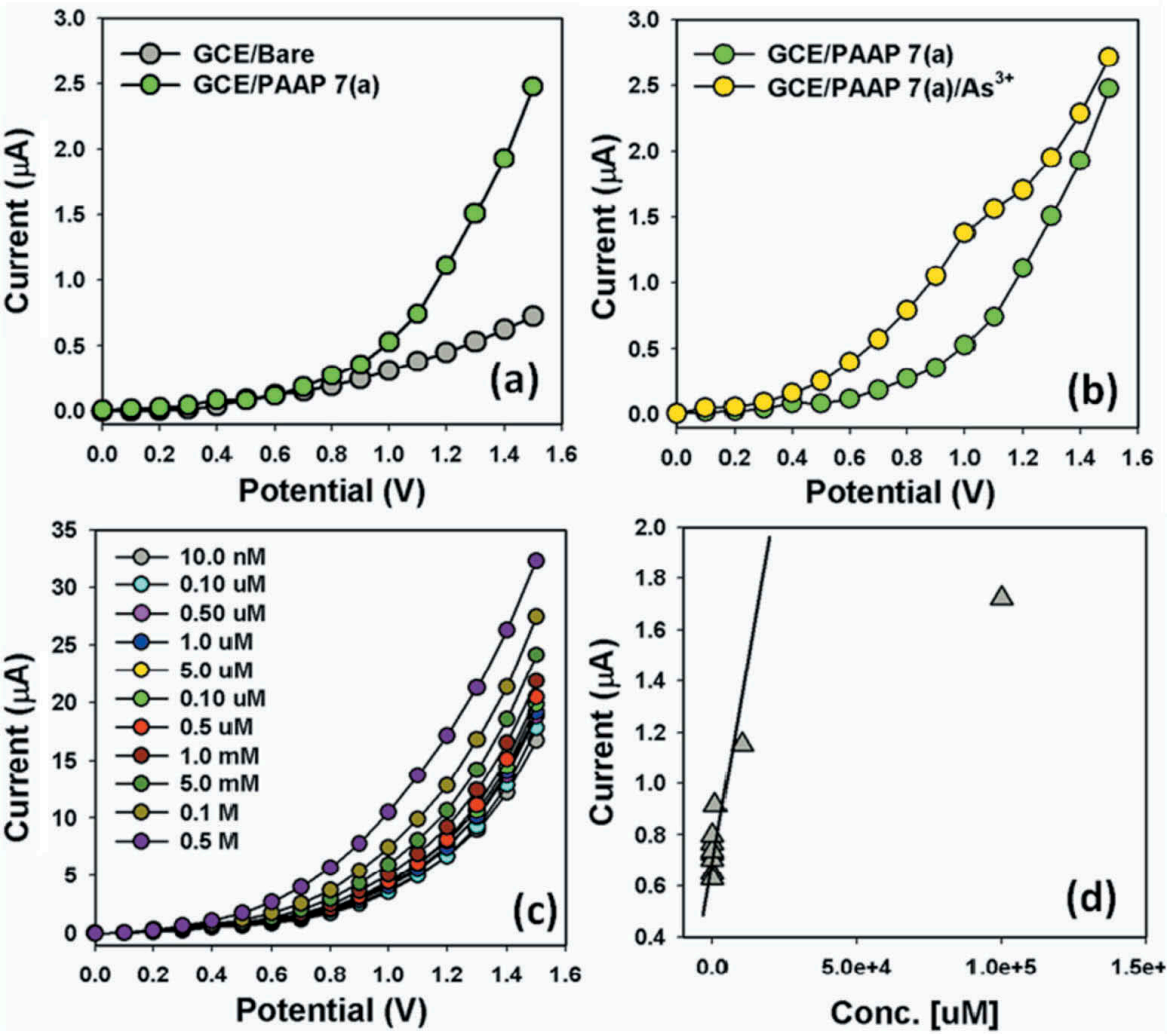
I-V responses of (a) bare-GCE and PAAP-coated/GCE; (b) PAAP/GCE (in absence and presence of As^3+^ ions) in the solution system. I-V responses of (c) concentration variations (10.0 nM ~ 0.5 M) of As^3+^ ions and (d) calibration plot of PAAP fabricated GCE electrode (at +0.5V).

The PAAP/Nafion/GCE was employed for the detection of As^3+^ ion in liquid phase. I-V responses were measured with PAAP/GCE coated thin-film (in two electrodes system). The concentration of As^3+^ ion was varied from 10.0 nM to 0.5 M by adding de-ionized water at different proportions. It is studied the control experiment about the uncoated and PAAP-coated electrode using I-V method and presented in Figure 8. Here, Figure 8(a) is represented the I-V responses for bare-GCE (grey-dotted) and PAAP/GCE (green-dotted) electrodes. In PBS system, the PAAP/GCE electrode shows that the reaction is enhanced slightly due to the presence of PAAP coated onto GCE surface. A considerable enhancement of current value with applied potential is demonstrated with fabricated PAAP/GCE in presence of target As^3+^ ion analyte, which is presented in Figure 8(b). The green-dotted and blue-dotted curves were indicated the response of the fabricated film after and injecting 25.0 µL As^3+^ ion into 5.0 mL PBS solution respectively measured by fabricated PAAP/GCE films. Significant increases of current are measured after every injection of target component in regular interval. I-V responses to varying Cu^2+^ concentration (10.0 nM to 0.5 M) on thin PAAP/GCE were investigated (time delaying, 1.0 sec) and presented in the Figure 8(c). Analytical parameters (such as sensitivity, detection limit, linearity, and linear dynamic range etc) were investigated from the calibration curve (current vs. concentration), which was presented in Figure 8(d). A wide range of As^3+^ concentration was selected to study the possible detection limit (from calibration curve), which was examined in 10.0 nM to 0.5 M. The sensitivity was calculated from the calibration curve, which was close to 2.714 µAcm^−2^µM^−1^. The linear dynamic range of the PAAP/GCE sensor was employed from 10.0 nM to 0.1 M (linearly, r^2^ = 0.9923), where the detection limit was calculated about 6.8 ± 0.1 nM (ratio, ^3N^/_S_). In presence of PAAP/Nafion/GCE layer on electrode, the electrical resistance is decreased under with the target As^3+^ in PBS phase. The film-resistance was decreased gradually (increasing the resultant current) upon increasing the As^3+^ concentration in bulk system.

In two-electrode system, I-V characteristic of the PAAP/GCE is activated as a function of As^3+^ concentration at room conditions, where the improved current response is observed. As obtained, the current response of the PAAP/Nafion/GCE is increased with the increasing concentration of As^3+^; however similar phenomena for cationic detection have also been reported earlier [59–61]. For a low concentration of As^3+^ in liquid medium, there is a smaller surface coverage of As^3+^ ions on PAAP/GCE film and hence the surface reaction proceeds steadily. By increasing the As^3+^ concentration, the surface reaction is increased significantly (gradually increased the response as well) owing to large surface area contacted with As^3+^ molecules. Further increase of As^3+^ concentration on PAAP/GCE surface, it is exhibited a more rapid increased the current responses, due to larger surface covered by As^3+^ chemical. Usually, the surface coverage of As^3+^ molecules on PAAP/GCE surface is reached to saturation, based on the regular enhancement of current responses. Selectivity was studied for sensor in presence other chemicals like Mg^2+^, Sn^2+^, As^3+^, Au^3+^, Al^3+^, Zn^2+^, Ce^2+^, Sb^3+^, Co^2+^, Cd^2+^, Ba^2+^, Ca^2+^ and Y^3+^ using the PAAP/Nafion/GCE, which is presented in Figure 5(a). It was observed the highest current response for As^3+^ by I-V measurement compared to other cations in the solution. The concentrations of heavy metallic analytes are kept constant at 0.1 µM level in PBS system. Therefore, As^3+^ was selectively detection with PAAP/GCE surface in this approach.

**Figure 9.**
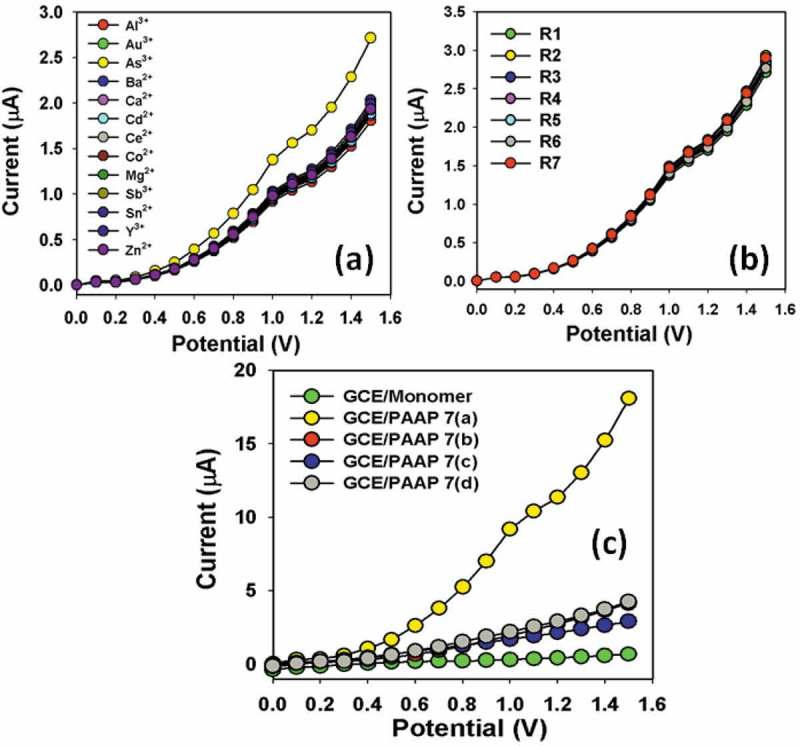
(a) Selectivity study with various cationic components by PAAP/GCE electrodes. (b) I-V responses of all reproducible signals (Run-1 to Run-7), and (c) various compositions of R (7a, 7b, 7c, and 7d) in PAAP. Analyte concentration was taken at 0.1µM. Potential range: 0 to +1.5V; Delay time: 1.0 sec.

To check the reproducibly and storage stabilities, I-V response for PAAP/GCE sensor was examined and presented in Figure 9(a). After each experiment (each runs), the fabricated PAAP/GCE substrate was washed thoroughly with the phosphate buffer solution and observed that the current response was not significantly decreased. Here it is observed the current loss in each reading is negligible compared to initial response of sensors using PAAP/GCE. A series of seven successive measurements of 0.1µM As^3+^ in 0.1 M PBS yielded a good reproducible signal at PAAP/GCE sensor with a relative standard deviation (RSD) of 2.58%. The sensitivity was retained almost same of initial sensitivity up to seven days, after that the response of the fabricated PAAP/GCE electrode gradually decreased. The As^3+^ sensor based on PAAP/GCE is displayed good reproducibility and stability for over week and no major changes in sensor responses are found. After a week, the chemical sensor response with PAAP/GCE was slowly decreased, which may be due to the weak-interaction between fabricated PAAP/GCE active surfaces and As^3+^ ions. I-V responses were also measured for all derivatives of R in PAAP polymer embedded GCE electrodes, such as 7(a), 7(b),7(c), and 7(d). It is observed that the current response was exhibited significantly higher for PAAP [R: 7(a)] compared to other composition (Figure 9c) due to the enhancement of large surface area into the polymer. Based on the current responses on various compositions, the details heavy metallic As^3+^ sensor development is approached for PAAP/GCE [7(a)] in terms of analytical parameters such as sensitivity, detection limit, linearity, reproducibility, selectivity etc. The higher current response of the fabricated PAAP/Nafion/GCE could be attributed to the excellent absorption (porous surfaces in PAAP/binders/GCE) and adsorption ability, high catalytic-decomposition activity, and good biocompatibility of the conduction PAAP polymers.

The significant result was achieved by PAAP/GCE, which can be employed as proficient electron mediators for the development of efficient cationic sensors. Actually the response time was around 10.0 sec for the fabricated PAAP/GCE to reach the saturated steady-state level [62]. The higher sensitivity of the fabricated PAAP/GCE could be attributed to the excellent absorption (porous surfaces in PAAP/Nafion/GCE) and adsorption ability and high catalytic-activity of branches derivative of PAAP. The estimated sensitivity of the fabricated sensor is relatively higher and detection limit is comparatively lower than previously reported cationic sensors based on other nano-composites or nano-materials modified electrodes measured by I-V systems [63–66]. Due to high specific surface area, PAAP provides a favorable nano-environment for the As^3+^ detection with good quantity. The high sensitivity of PAAP/Nafion/GCE provides high electron communication features which enhanced the direct electron transfer between the active sites of PAAP and coated-GCE. The PAAP/GCE system is demonstrated a simple and reliable approach for the detection of toxic heavy cationic species. It is also compared the performance of the PAAP/Nafion/GCE electrode for the selective detection of As^3+^ depending on various compounds or materials by electrochemical approaches [67–78] and compared to other published reports and presented in Table 3 As per the observation, the sensor probe of PAAP/Nafion/GCE exhibited the highest sensitivity compared with other values. It is also revealed that the significant access to a large group of cations for wide-range of ecological, environmental and health-care fields.

**Table 3.** Comparison the sensor performances towards As^3+^detection based on various compounds or materials used and measured by electrochemical approaches.

Electrode	Method	Sensitivity (μAμM^−1^cm^−2^)	LOD	LDR	Ref.
Au-NPs/aptamer	CM + RS	–	0.6 ppb	1–1500 ppb	[66]
Au-NPs/PC_3_R	CM	–	20	–	[67]
Substrate	RRS	–	0.2 ppb	0.1 ppb-200 ppb	[68]
ITO	ECC	–	1.2 μM	–	[69]
CdSe/ZnS QDs	–	–	5.0 μM	–	[70]
Apt-SNPs	CM	–	6.0 μg L^−1^	–	[71]
AgNPs/GO/GCE	SW-ASV	0.1805	0.24	13.33–375.19	[72]
PtNPs/GCE	SW-ASV	0.00022	26.7	1000–50,000	[73]
Ir/BBDE	CV	0.00703	20.0	93–9800	[74]
AgNPs/CT/GCE	DPASV	0.000308	16.0	130–13,300	[75]
Au nano-array/GCE	ASV	0.00091	80.0	9800–59,200	[76]
Nano-Pt-Fe(III)/MWCNT/GCE	ASV	0.00476	10.0	100–10,000	[77]
AuNP/GCE	LSV	0.0142	1.0	–	[78]
PAAP/Nafion/GCE	**I-V**	**2.714**	**6.8 ± 0.1 nM**	**10.0 nM to 0.1 M**	**Thiswork**

CM: Colorimetric, RS: Resonance scattering, PC_3_R: (γ-Glu-Cys)_3_-Gly-Arg, RRS: Resonance Rayleigh Scattering, ECC: Electrochemical-chemical-chemical, ITO: Indium-Tin-Oxide, CV: Cyclic Voltametry. I-V: Current vs. Voltage method.

The real samples such as industrial effluent, tape and sea water were analyzed in order to validate the proposed I-V technique using PAAP/Nafion/GCE for arsenic determination. A standard addition method had been applied to estimate the concentration of As^3+^ in real samples. A fixed amount (~25.0 µL) of each real sample was analyzed in 10.0 mL bulk solution (PBS, 0.1 M) using the fabricated PAAP/Nafion/GCE. The results have been included regarding the measurement of As^3+^ in industrial effluent, tap and sea water samples, which apparently confirmed that the proposed I-V technique is satisfactory, reliable, and suitable for analyzing real samples with assembled of PAAP/Nafion/GCE (Table 4).

**Table 4.** Measured As^3+^ concentration in different real samples.

Real samples	Calibrated concentration range	Measured current (μA)	Respective concentration (nM)
Industrial effluent		5.1	~0.782 ± 0.01
Tap water	10.0 nM ~ 0.1 M	1.9	~0.092 ± 0.01
Sea water		1.6	~0.085 ± 0.01

## 4. Conclusion

Thermally stable hybrid polyarylidene(azomethine-ether)s and copolyarylidene-(azomethine – ether)s containing diarylidenecycloalkanone moieties as cornerstones are synthesized by solution poly-condensation method. The required Monomers, pre-monomers and model compound are also synthesized and their chemical framework is established by right elemental and spectral analyses. Variable characterization tools are also tested and are studied for the desired compounds. All the synthesized polymers and copolymers are completely insoluble in most simple organic solvents. The PDT thermal stabilities for all the tested polymers and copolymers are in the order **7_c_ > 8_d_ > 8_e_ > 8_a_ > 8_b_ > 7_a_**. Although polymer **7_a_** is considered as the lower thermally stable polymer at T_10_, T_25_ and T_50_, even it still has higher FDT value like the other stable derivatives. Furthermore, all the polymers and copolymers have high *T_g_* values except for polymer **7_a_** which has a distinct weak value (57.08°C). Later, PAAP was fabricated for detecting selective cation with the conducting coating binders onto flat GCE by electrochemical approaches, which displayed higher sensitivity and selective towards As^3+^ ions. The analytical performances of the fabricated As^3+^ sensors are excellent in terms of sensitivity, detection limit, linear dynamic ranges, selectivity, and in short response time. PAAP/Nafion/GCE assembly is exhibited higher-sensitivity 2.714 µAcm^−2^µM^−1^) and lower-detection limit (6.8 ± 0.1 nM) with good linearity in short response time, which efficiently utilized as cationic-sensor for selective As^3+^ onto PAAP/Nafion/GCE. This novel approach is introduced a well-organized route of efficient heavy metallic sensor development for environmental pollutants and health-care fields in broad scales.
